# Effects of varying longitudinal bending stiffness in running shoes on lower limb biomechanics of elite marathon runners

**DOI:** 10.3389/fspor.2025.1608092

**Published:** 2025-05-27

**Authors:** Hailiang Yang, Yuhui Cai, Kuang Li, Xiangdong Wang, Tao Liu

**Affiliations:** ^1^ School of Physical Education, Jimei University, Xiamen, Fujian, China; ^2^ Footwear Science & Technology Innovation Center, Xtep (China) Co., Ltd., Xiamen, Fujian, China

**Keywords:** marathon shoes, carbon fiber plate, longitudinal bending stiffness, elite runners, biomechanics, rearfoot-strike pattern

## Abstract

**Objective:**

This study examined the effect of three levels of longitudinal bending stiffness (LBS) in carbon-plated shoes on lower limb biomechanics.

**Methods:**

Fifteen elite male marathon runners, each with a personal best under 3 h, participated in the study. They were tested wearing shoes with three LBS levels: low (LLBS, 0.31 Nm/deg), medium (MLBS, 0.40 Nm/deg), and high (HLBS, 0.48 Nm/deg). All participants ran at a constant speed of 4.76 m/s. Kinematic and kinetic data were synchronously collected using the VICON motion capture system and three AMTI force plates. Angular parameters of the ankle, knee, hip, and metatarsophalangeal (MTP) joints were calculated using the sagittal plane coordinate system. Joint moments and joint work (positive and negative) at each lower limb joint were analyzed using the built-in inverse dynamics module in Visual3D.

**Results:**

In terms of kinematics, the maximum dorsiflexion angle of the MTP joint during the late stance phase and the range of motion during the stance phase was significantly lower in the MLBS to HLBS shoes than in the LLBS shoes. Negative work at the MTP joints was significantly higher in the LLBS shoes than in the HLBS shoes (LLBS: 0.21 ± 0.05 J/kg, MLBS: 0.16 ± 0.03 J/kg, HLBS: 0.13 ± 0.05 J/kg, *F* = 12.053, *p* = 0.001). Additionally, the maximum hip extension moment was also significantly affected by LBS (F = 6.561, *p* = 0.012, *η*2 = 0.286), with the MLBS (1.49 ± 0.27 Nm/kg) shoes showing lower values than both LLBS (1.80 ± 0.33 Nm/kg, *p* = 0.021) and HLBS (1.58 ± 0.32 Nm/kg, *p* = 0.047). The MLBS shoes significantly increased positive work at the ankle joint (LLBS: 0.66 ± 0.08 J/kg, MLBS: 0.71 ± 0.10 J/kg, HLBS: 0.69 ± 0.08 J/kg, *F* = 3.292, *p* = 0.047).

**Conclusions:**

This study demonstrates that increasing the LBS shoes alters the lower limb biomechanical performance of elite marathon runners. Both MLBS and HLBS conditions reduced MTP joint dorsiflexion, but MLBS shoes significantly increased positive work at the ankle joint and improved joint function while maintaining lower hip joint moments. Among the three conditions, MLBS better balanced mechanical efficiency and natural joint function, suggesting it may be a more biomechanically suitable option for elite marathon runners. These findings provide valuable insights for optimizing LBS in performance shoe design, with MLBS offering a potential advantage in both biomechanical performance and injury prevention.

## Introduction

Marathon events surged in popularity worldwide beginning in the 1970s and 1980s, (1–4). As marathon sports rapidly developed, the marathon footwear industry expanded accordingly, with manufacturers continuously introducing new running shoe models. In response to the pioneering “Nike Vaporfly 4%,” which demonstrated significant performance benefits for marathon running, numerous footwear companies have developed advanced footwear technologies (AFT) (5). AFT shoes feature a curved rigid plate embedded within a soft, highly resilient foam midsole, combined with a distinct sagittal-plane rocker sole geometry (6). The integration of a curved rigid carbon plate elevates the longitudinal bending stiffness (LBS) of shoes, consequently inducing a series of kinematic and kinetic alterations in a runner's gait. These modifications include increased ankle dorsiflexion, reduced metatarsophalangeal(MTP) dorsiflexion, and an increased moment arm of the ground reaction force (GRF) (7–9).

Variations in carbon fiber plate(CFP) geometry and placement induce distinct modifications in lower-extremity biomechanical parameters during running. Song et al. (10) demonstrated through finite element analysis that embedding a 3 mm carbon plate above the outsole reduced peak plantar pressure during forefoot strike by 31.91% compared with non-plated shoes. Additional research (11) found that curved CFP provide forefoot pressure reduction (5.51%–12.62% greater attenuation) superior to flat plate configurations. Flore et al. (12) positioned the CFP between the insole and midsole, observing reductions in vertical ground reaction force (GRF) and positive work at the knee joint during running. McLeod et al. (13) examined two running speeds (2.98 vs. 4.47 m/s) and found that rearfoot strikers required higher LBS at faster speeds, whereas midfoot strikers maintained optimal LBS across speeds. This discrepancy originates from their distinct movement patterns: rearfoot strikers depend on LBS for propulsion, whereas midfoot strikers complete the push-off motion through MTP joint movement.

In March 2025, the Chinese Athletics Association issued the China Athletics Association Road Race Classification Standards for Amateur Runners (Implementation Measures), defining amateur runners under 34 years old who complete a full marathon within 3 h as “Elite Level” (14). Among amateur runners, those achieving full marathons under three hours represent a unique cohort, combining high participation enthusiasm with near-professional performance. This group exhibits significantly higher weekly running volumes (≥50 km) and joint loading intensities than average runners (15), yet demonstrates smaller Achilles tendon cross-sectional areas than elite athletes (16). When wearing shoes with varying LBS, they may display distinct lower-limb biomechanical responses. Moreover, discrepancies in CFP placement between experimental shoe designs and commercially available models complicate the direct application of existing findings to product optimization (17–19). Some studies place CFP between the midsole and insole, contrary to commercial marathon shoes, which embed them within the midsole. These differences cause difficulty to directly apply the conclusions of such studies to shoes (17–19).

A variety of mechanistic explanations have been proposed for the effects of LBS on lower extremity biomechanics during running. Some scholars have suggested that an increase in LBS may limit excessive dorsiflexion of the MTP joints, which in turn reduces energy loss and improves ankle joints positive work (7, 20). Some studies have also observed differences in the distribution of joint power and moments under different stiffness conditions, but there are no consistent conclusions suggesting that increased stiffness systematically alters the proportion of loading on the proximal or distal joints (21, 22). The shoe designs used in these studies varied widely, including the location (above the insole, inside the midsole, or close to the outsole) and shape (flat or curved plate) of the carbon plate, making the findings difficult to compare (23, 24). In addition, most of the current studies on the effects of LBS have focused on average runners, covering subject groups with large differences in training intensity, gait patterns, and joint control (25). Elite-level marathon runners, with long-term training accumulation and relatively stable movement patterns, may be unique in terms of power chain coordination, energy utilization patterns, and response mechanisms to external structures (26). Therefore, it is difficult to directly extrapolate the conclusions drawn from a sample of mass runners only to the elite runner population. In the existing literature, there is still a lack of systematic research on the biomechanical performance of the lower limbs of elite runners wearing commercially available LBS shoes at real race speeds, especially in the rearfoot strike mode, where the specific effects of different LBS settings on the dynamic characteristics of the joint angles, moments, and work done are still unclear.

Therefore, this study aims to examine the effect of shoes with varying LBS levels on lower-limb biomechanics in runners who complete a full marathon within 3 h using a rearfoot strike pattern. We hypothesize that higher LBS will decrease maximum MTP joint dorsiflexion angles and negative work, at the same time increasing positive work at the MTP and ankle joints. To test this theory, we analyze kinematic and kinetic data from male rearfoot-strike runners (full marathon times under 3 h), using shoe designs that replicate commercial models with midsole-embedded CFP. If validated, these findings may provide scientific guidance on shoe selection advice for elite marathon runners and serve as a reference for optimizing LBS design in marathon shoes.

## Methods

### Participants

The sample size was calculated using G*POWER software (Erdfelder, Faul, & Buchner, 1996). The actual statistical analysis method used was repeated-measures analysis of variance (ANOVA), with an effect size of 0.4, a significance level (α) of 0.05, a power (1-β) of 0.8, one group, and three measurements. The assumed effect size (*f* = 0.4) was informed by prior studies reporting substantial biomechanical differences across shoe stiffness conditions, supporting the justification for a large effect (20, 21, 27). The minimum required sample size was 12. Ultimately, fifteen healthy adult male marathon runners were recruited. The inclusion criteria were as follows: European shoe size 41, right leg dominance, rearfoot strike pattern, no history of lower limb neuromuscular injuries in the past three years, a weekly running distance of not less than 50 km, and a marathon personal best of under 3 h.

### Experimental shoe design

**Figure 1:**
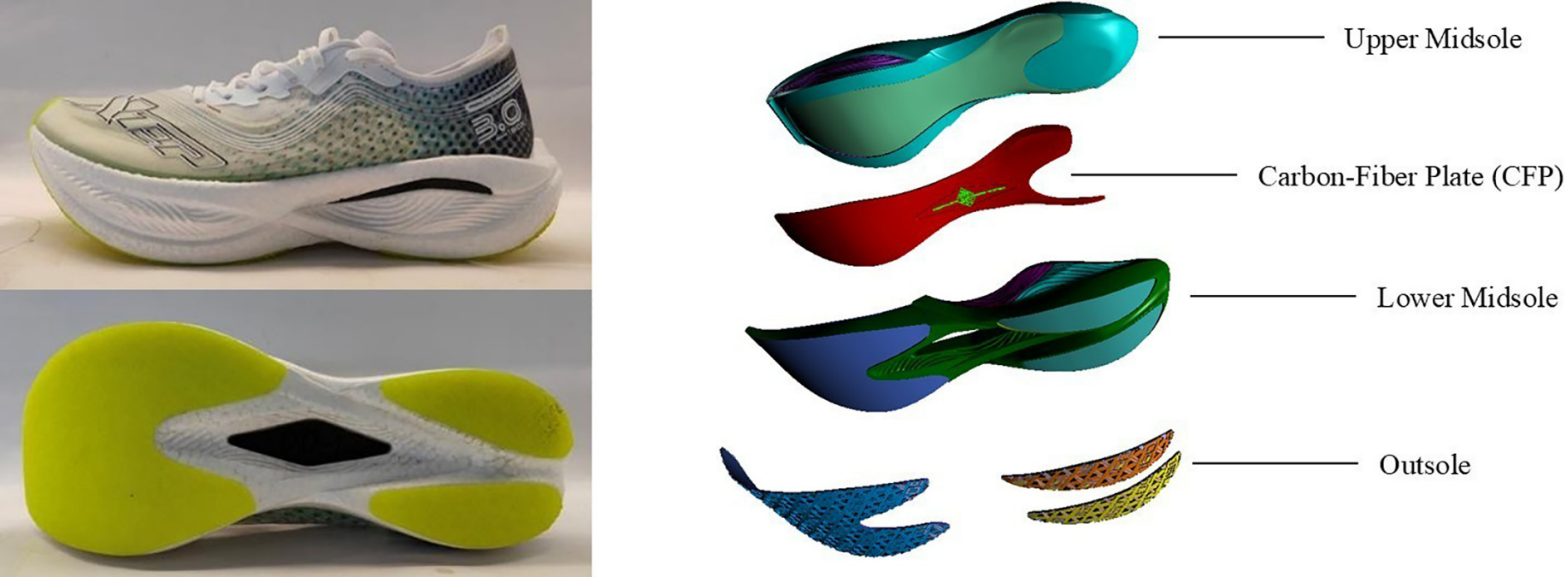
External appearance of the experimental shoe and structural illustration of the embedded carbon fiber plate.

The experimental footwear for this study was provided by Xtep Co., Ltd. (China), using the Xtep 160X 3.0 Pro running shoe as the base model, with modifications not available in commercial models. The shoes featured a curved CFP embedded within the midsole, as illustrated in Figure 1. The shoes were categorized into three LBS conditions based on the thickness of the CFP: LLBS (no CFP), MLBS (1.0 mm CFP), and HLBS (1.4 mm CFP). The other components of the shoes, such as the outsole construction and materials, upper design, and shoelace configuration, remained completely identical. Mechanical testing was conducted on the forefoot region using a bending stiffness tester to quantify LBS (C4D17NZ53A, Exeter Research, USA). The measured LBS values for the three shoe conditions were as follows: LLBS 0.31 Nm/deg, MLBS 0.40 Nm/deg, and HLBS 0.48 Nm/deg. All experimental shoes maintained identical structural configurations, materials, and uppers, with the sole variation being the bending stiffness (LBS) resulting from carbon plate integration. The weights of the three pairs of shoes were 221.77 g (LLBS), 219.83 g (MLBS), and 227.08 g (HLBS).

### Experimental procedure

A 10-camera VICON motion capture system (200 Hz, Oxford Metrics Ltd., UK) synchronized with three AMTI force plates (1,000 Hz, AMTI, Watertown, MA, USA) via VICON Nexus software and Lock + hardware module was used to collect kinematic and kinetic data. Data were collected as participants ran at a controlled speed while wearing three experimental shoes with LBS levels in a laboratory setting. Running speed (4.76 m/s) was strictly monitored using Smartspeed timing gates (Fusion Sport International, Coopers Plains, AUS), with a permitted deviation of ±5% (24, 28). Two timing gates were positioned four meters apart, with three force plates arranged between them. The length of the laboratory runway is 60 meters with a concrete surface.

**Figure 2:**
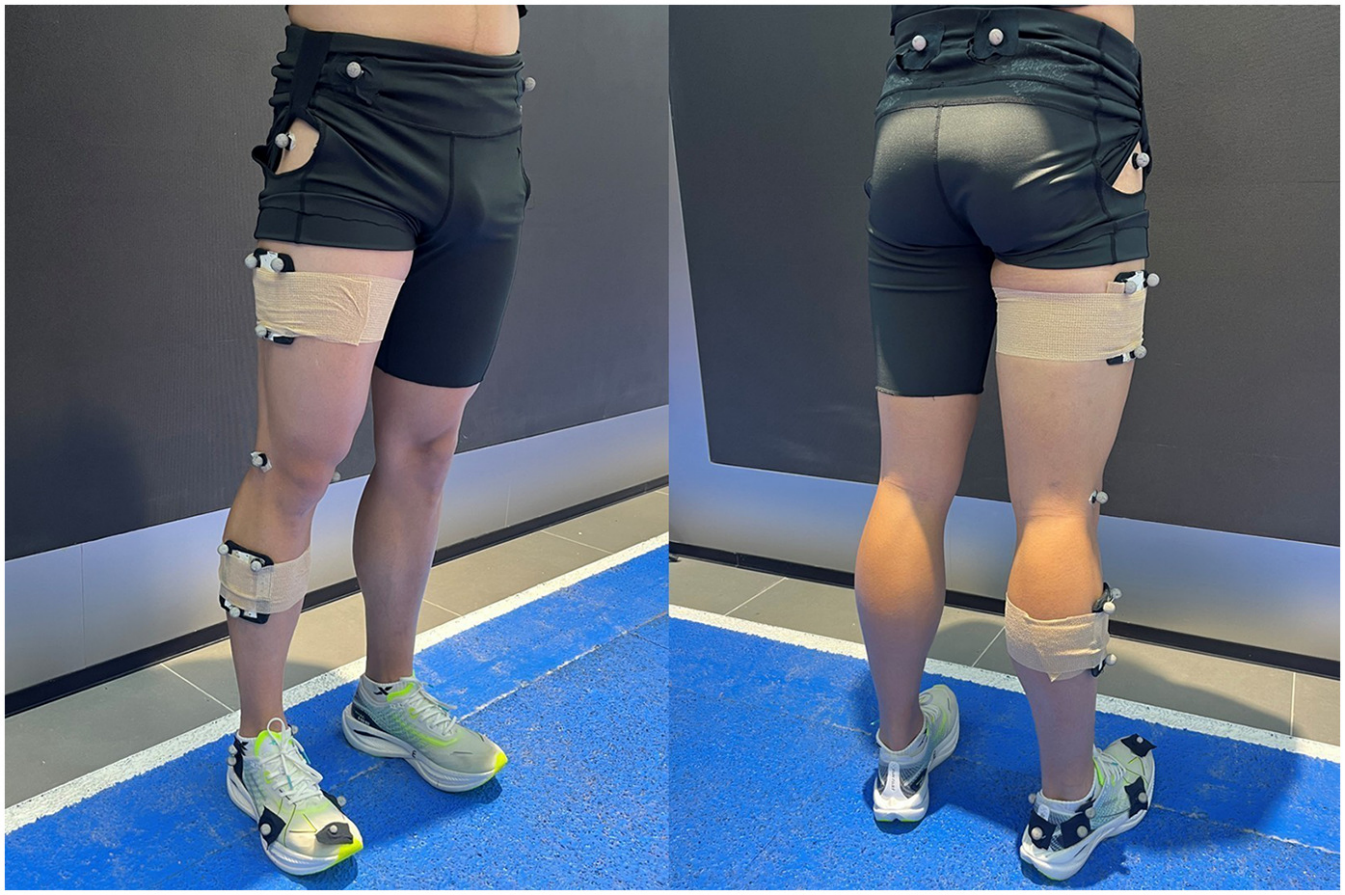
The lower extremity reflective marker Set.

Prior to the experiment, participants signed informed consent forms, and their height and body mass were measured for subsequent individualized biomechanical modeling parameter input. Following a 10-minute warm-up run for environmental acclimation, 21 reflective markers (14 mm diameter) were attached to anatomical landmarks: left anterior superior iliac spine, right anterior superior iliac spine, left posterior superior iliac spine, right posterior superior iliac spine, as well as unilaterally to the right greater trochanter, medial and lateral femoral epicondyles, and medial and lateral malleoli. A three-marker tracking cluster was placed on the lateral aspect of the thigh, and another three-marker cluster was affixed to the lateral aspect of the shank (29). Additional markers were attached to the shoe overlying the first and fifth metatarsal heads to define the foot segment, as shown in Figure 2 (30, 31). One reflective marker was affixed to the anterior tip of the shoe, above the second toe. Furthermore, shoe-mounted tracking markers were attached to the distal and proximal posterior heel, as well as to the lateral rearfoot. The experimental trials followed a randomized design, where each participant ran under three different experimental shoe conditions (LLBS, MLBS, and HLBS). A minimum of five valid trials were collected under each condition per participant. Participants were instructed to maintain natural running posture, with the right foot fully contacting the force plate during stance phase and maintaining speed deviations within 5% of the preset value for valid trial acquisition. A trial was considered valid only when the participant's right foot fully contacted the central active sensing area of the force plate during stance, avoiding heel or toe strikes outside the perimeter. Adequate rest intervals were maintained between successive footwear testing sessions to mitigate fatigue accumulation. Experimental investigators continuously monitored participants' fatigue status throughout the testing protocol. Participants who self-reported fatigue during trials were permitted to pause testing until fully recovered.

### Data processing and analysis

This study conducted a biomechanical analysis focused on the stance phase of running, defined as the period from right heel contact to toe-off of the distal phalanx of the right second toe. Raw data were exported as C3D files from Vicon Nexus and processed using the Visual3D (C-Motion Inc., USA). Heel contact was identified when vertical GRF exceeded 15N, whereas toe-off was defined when force decreased below 15 N (32). To eliminate signal noise, a fourth-order zero-phase Butterworth filter was applied, with cutoff frequencies set at 10 Hz for kinematic data and 50 Hz for kinetic data (33). Angular parameters for the ankle, knee, hip, and first MTP joints were calculated using the sagittal plane coordinate system. Joint moments and power were determined through the inverse dynamics model integrated into the software, while normalizing the joint dynamics based on body mass (34). We extracted: (1) maximum MTP dorsiflexion, ankle plantarflexion, knee extension, hip extension angles during late stance phase; (2) MTP, ankle, knee, and hip joints ROM in the sagittal plane during stance phase; (3) Maximum joint moments in the sagittal plane at the MTP, ankle, knee, and hip joints during the stance phase; (4) Positive and negative joint work at the MTP, ankle, knee, and hip joints during the stance phase as independent variables. Joint angles, angular velocities, and joint moments of the dominant lower limb (hip, knee, ankle, and MTP joints) were calculated using the built-in inverse dynamics module of the Visual3D software (C-Motion, Inc., USA). Joint power was obtained as the product of joint moment and angular velocity. Positive and negative joint work were computed by integrating the joint power curve over time. Power values above the horizontal axis were defined as positive, while those below were defined as negative (24).

### Statistical methods

All statistical analyses in this study were performed using SPSS 27.0 (IBM Corp., USA). Continuous variables were expressed as mean ± standard deviation (mean ± SD) after testing for normality using the Shapiro–Wilk test. A one-way repeated measures ANOVA was used to assess the effect of shoe stiffness on biomechanical parameters. When the Mauchly's test of sphericity indicated a violation of the sphericity assumption, the Greenhouse–Geisser correction was applied to adjust the degrees of freedom. *post-hoc* multiple comparisons were conducted using the Bonferroni correction, and statistical significance was defined as *p* < 0.05. Standardized effect sizes for assessing the strength of main effects in ANOVA were assessed using partial *η*² (partial eta-squared). According to the empirical guidelines proposed by Cohen, the effect strengths of partial *η*² were classified as follows: 0.01 ≤ *η*^2^ < 0.06 for small effects, 0.06 ≤ *η*^2^ < 0.14 for medium effects, and *η*^2^ ≥ 0.14 for large effects (35).

## Results

Fifteen healthy adult male marathon runners were recruited as participants (age: 26.2 ± 4.2 years, mass: 60.16 ± 5.22 kg, height: 169.55 ± 4.77 cm, and BMI: 21.54 ± 1.70 kg/m²). The Shapiro–Wilk test showed that all indicators followed a normal distribution (*p* > 0.05).

### Kinematic results

**Figure 3:**
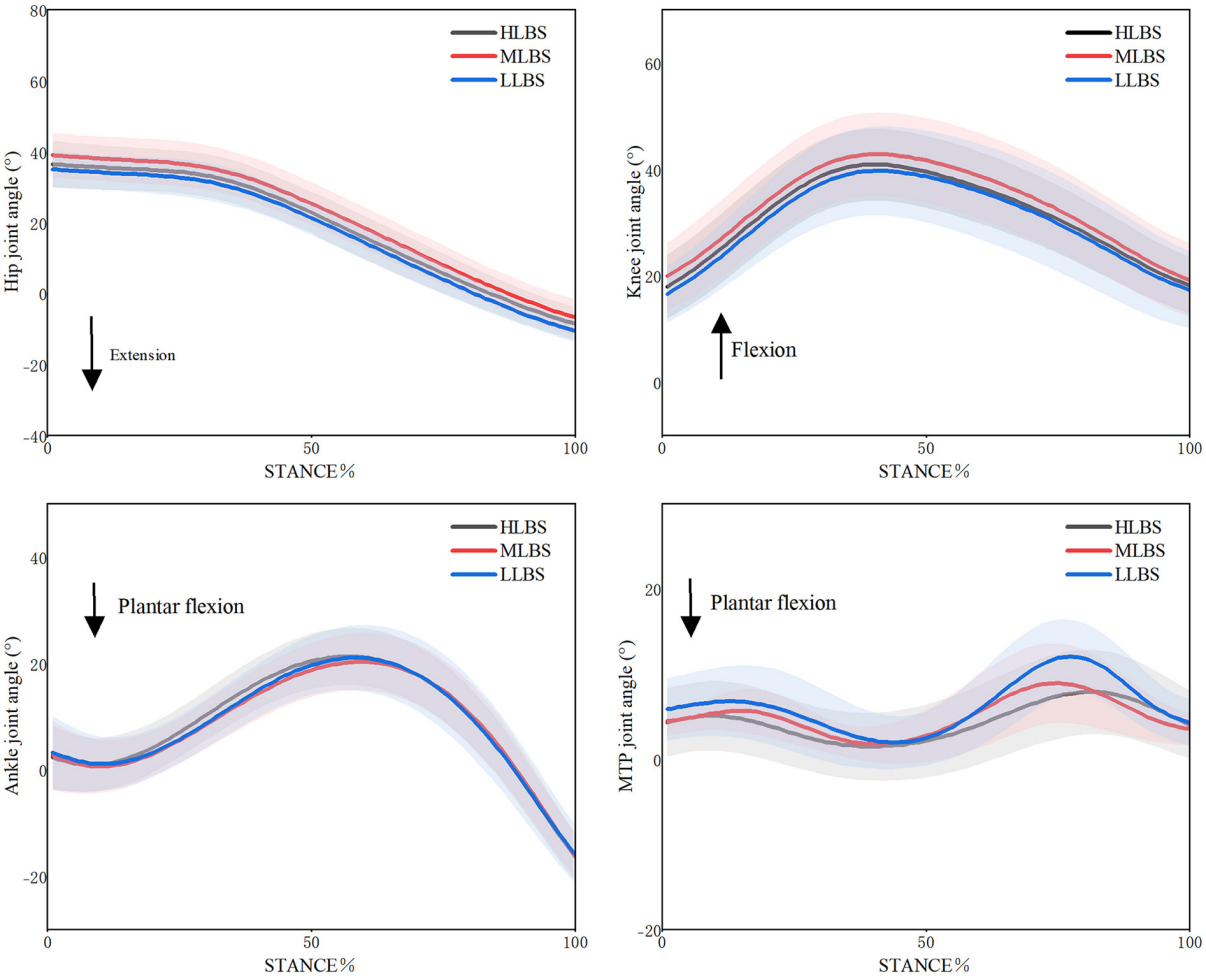
Variations in sagittal plane joint angles of the lower extremity under LLBS, MLBS, and HLBS conditions.

The maximum joint angles during late stance phase and the range of motion(ROM) during stance phase of each lower limb joint under different LBS conditions are shown in Table 1 and Figure 3, respectively. The main effect of maximum hip extension angle was significant (Wilks' Lambda = 0.40, *F* = 3.669, *p* = 0.46, *η*^2^ = 0.290), and *post-hoc* multiple comparisons revealed that the LLBS was significantly higher than the MLBS (*p* < 0.01). The main effect of the maximum dorsiflexion angle of the MTP joint during late stance phase was also significant (Wilks' Lambda = 0.48, *F* = 10.828, *p* < 0.01, *η*^2^ = 0.436). *post-hoc* multiple comparisons demonstrated that the LLBS shoe had a significantly higher maximum dorsiflexion angle than the MLBS (*p* = 0.0335) and the HLBS (*p* = 0.004). Furthermore, the HLBS was significantly lower than the MLBS (*p* < 0.01). No significant main effects or *post-hoc* multiple comparisons were observed for maximum knee extension angle or the maximum ankle plantarflexion angle during the late stance phase (*p* > 0.05).

**Table 1:** Mean ± standard deviation of kinematic outcomes in the sagittal plane for LLBS, MLBS, and HLBS shoes.

Joint	Maximum Angle(°) during late stance Phase			Range of Motion(°) during Stance Phase		
	LLBS	MLBS	HLBS	LLBS	MLBS	HLBS
Hip joint	−12.97 ± 2.65	−11.06 ± 3.70^a^	−12.16 ± 3.67	46.03 ± 4.95	46.14 ± 4.40	45.41 ± 4.40
Knee joint	23.10 ± 6.26	23.86 ± 5.50	23.08 ± 4.50	24.94 ± 3.38	25.17 ± 3.25	24.90 ± 2.72
Ankle joint	−18.50 ± 3.83	−18.78 ± 3.29	−19.18 ± 3.65	38.32 ± 3.07	38.00 ± 3.12	39.09 ± 3.33
MTP joint	14.00 ± 3.69	11.76 ± 3.09^a^	10.62 ± 2.71^ab^	11.63 ± 3.06	9.19 ± 2.45^a^	7.78 ± 1.77^a^

Note. Negative value indicates extension/plantarflexion; Positive value indicates flexion/dorsiflexion.

^a^ indicates significantly difference from LLBS.

^b^ indicates significantly difference from MLBS.

The ROM of the MTP joint during the stance phase was statistically significant based on the sphericity test (Wilks' Lambda = 0.24, *F* = 25.478, *p* < 0.01, *η²* = 0.560). The ROM in the LLBS condition was significantly greater than that in both the MLBS (*p* < 0.01) and HLBS conditions (*p* < 0.01). No significant main effects or *post-hoc* differences were found for the ROM of the other lower limb joints (*p* > 0.05).

### Kinetic results

**Figure 4:**
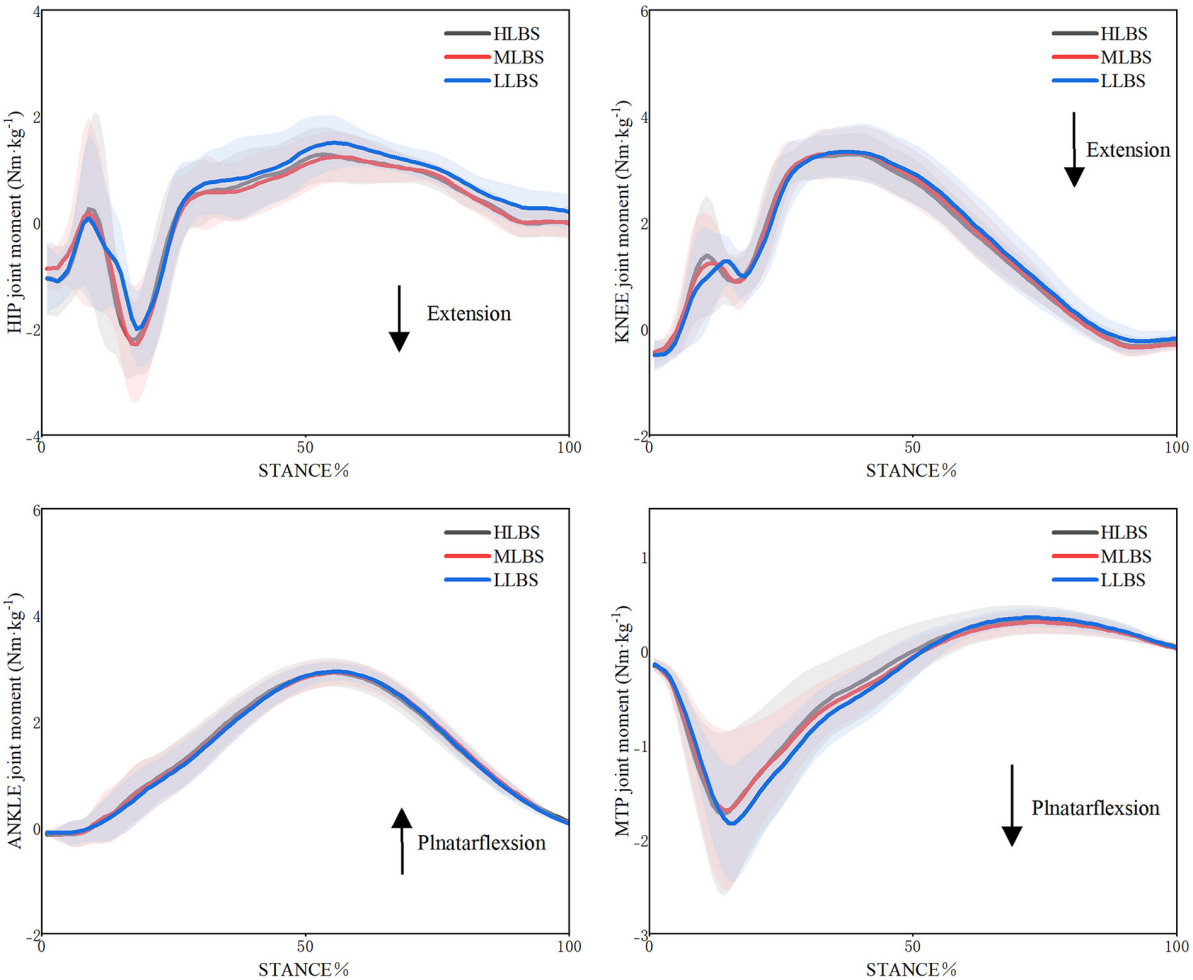
Variations in sagittal plane joint moments of the lower extremity under LLBS, MLBS, and HLBS conditions.

The joint moments in the sagittal plane for LLBS, MLBS, and HLBS shoes are presented in Table 2 and Figure 4. The maximum hip extension moment was statistically significant after Greenhouse–Geisser correction (Wilks' Lambda = 0.48, *F* = 6.561, *p* = 0.012, *η²* = 0.286). The MLBS condition (1.49 ± 0.27 Nm/kg) exhibited significantly lower values than both LLBS (1.80 ± 0.33 Nm/kg, *p* = 0.021) and HLBS (1.58 ± 0.32 Nm/kg, *p* = 0.047).

**Table 2:** Mean ± standard deviation of joint moments in the sagittal plane for LLBS, MLBS, and HLBS shoes.

Joint	Maximum joint moment (Nm/kg) during middle to late stance phase		
	LLBS	MLBS	HLBS
Hip joint	1.80 ± 0.33	1.49 ± 0.27^a^	1.58 ± 0.32^ab^
Knee joint	2.95 ± 0.56	2.88 ± 0.58	2.87 ± 0.44
Ankle joint	2.97 ± 0.17	2.93 ± 0.23	2.93 ± 0.27
MTP joint	0.37 ± 0.06	0.34 ± 0.06	0.38 ± 0.13

Note. Positive value indicates extension/plantarflexion; Negative value indicates flexion/dorsiflexion.

^a^ indicates significantly difference from LLBS.

^b^ indicates significantly difference from MLBS.

**Figure 5:**
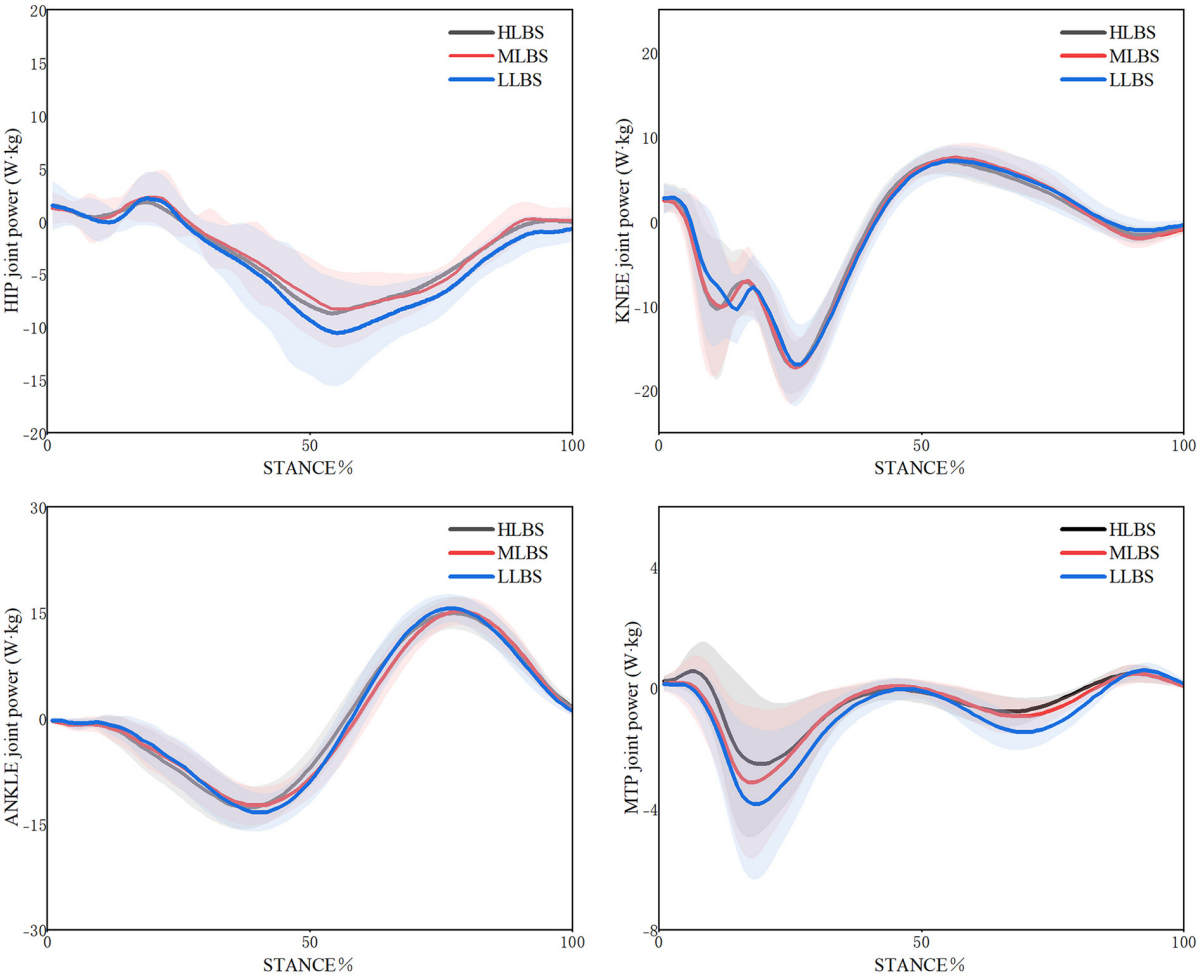
Variations in sagittal plane joint power of the lower extremity under LLBS, MLBS, and HLBS conditions.

The joint works in the sagittal plane for LLBS, MLBS, and HLBS shoes are summarized in Table 3 and Figure 5. A significant main effect was observed for the negative work of hip joint after Greenhouse–Geisser correction (Wilks' Lambda = 0.61, *F* = 5.774, *p* = 0.007, *η²* = 0.254), with the HLBS condition showing significantly lower values than LLBS (*p* = 0.016). No significant differences were found for negative work at the knee and ankle joints (*p* > 0.05). For the MTP joint, negative work also showed a significant main effect (Wilks' Lambda = 0.26, *F* = 12.053, *p* = 0.001, *η²* = 0.668), with HLBS yielding significantly lower values than LLBS (*p* = 0.019). A significant main effect was also found for positive work at the ankle joint based on the sphericity test (Wilks' Lambda = 0.38, *F* = 3.292, *p* = 0.047, *η²* = 0.141); *post-hoc* comparisons revealed that MLBS produced significantly greater values than LLBS (*p* = 0.039). Regarding the positive work at MTP joint, a significant main effect was detected (Wilks' Lambda = 0.27, F = 6.002, *p* = 0.008, *η*² = 0.232), with HLBS shoes showing significantly higher values than LLBS (*p* = 0.019).

**Table 3:** Mean ± standard deviation of joint works in the sagittal plane for LLBS, MLBS, and HLBS shoes.

Joint	Negative Work (J/kg)			Positive Work (J/kg)		
	LBS	MLBS	HLBS	LLBS	MLBS	HLBS
Hip Joint	−0.76 ± 0.22	−0.68 ± 0.10	−0.65 ± 0.16^a^	0.10 ± 0.02	0.11 ± 0.03	0.11 ± 0.02
Knee Joint	−0.61 ± 0.17	−0.63 ± 0.12	−0.62 ± 0.10	0.39 ± 0.12	0.38 ± 0.09	0.37 ± 0.09
Ankle Joint	−0.63 ± 0.10	−0.62 ± 0.10	−0.62 ± 0.12	0.66 ± 0.08	0.71 ± 0.10^a^	0.69 ± 0.08
MTP Joint	−0.21 ± 0.05	−0.16 ± 0.03	−0.13 ± 0.05^a^	0.02 ± 0.01	0.02 ± 0.01	0.03 ± 0.02^a^

^a^ indicates significantly difference from LLBS.

## Discussion

This study aims to reveal the influence of different LBS shoes on the lower limb biomechanical characteristics of rearfoot runners with a full marathon time under 3 h. It also seeks to provide scientific guidance for elite shoe selection and offer design references for LBS optimization in marathon footwear. The biomechanical data of the lower limbs of the subjects wearing three different LBS shoe types (0.31, 0.40, and 0.48 Nm/deg) were collected when they were running at a speed of 4.76 m/s. Selected lower extremity biomechanical indices included (1) maximum MTP dorsiflexion, ankle plantarflexion, knee extension, hip extension angles during late stance phase; (2) MTP, ankle, knee, and hip joints ROM in the sagittal plane during stance phase; (3) peak joint moments in the sagittal plane at the MTP, ankle, knee, and hip joints during the stance phase; (4) Positive and negative joint work at the MTP, ankle, knee, and hip joints during the stance phase as independent variables.

The results of the study revealed that the enhancement of the LBS of the shoes significantly altered the biomechanical performance of the lower limb joints during running. At the kinematic level, MLBS and HLBS shoes significantly reduced the maximum dorsiflexion angle of the MTP joints during late stance phase and the ROM during the stance phase. Moreover, the maximum hip extension angle was significantly reduced in the MLBS condition compared to the LLBS condition. Kinetic analysis showed that the hip extension moment decreased significantly with increasing LBS, with the greatest reduction observed in the MLBS condition. The HLBS shoes significantly reduced the negative work of the MTP joints and hip joints. Furthermore, enhancing the positive work of the MTP joints and the positive work of the ankle joints showed a compensatory increase with increasing LBS.

In this study, MLBS and HLBS shoes significantly reduced the maximum dorsiflexion angle of the MTP joints during late stance phase and the ROM during stance phase, highly consistent with the findings of Hoogkamer et al. (7). Hoogkamer's team compared the different stiffness levels of the shoes and found that HLBS shoes limit the maximum dorsiflexion angle of the MTP joints. This mechanism results from the rigid structure of high LBS shoes, reducing energy loss by limiting the dorsiflexion angle of the MTP joints. The LBS of shoes is beneficial to running energetics provided it does not interfere with the natural flexion of the MTP joints. This benefit may be diminished if the LBS is extremely high, limiting the normal motion of the MTP joints (8). In addition, the maximum hip extension angle during late stance phase was significantly reduced by 14.7% for the MLBS compared with the LLBS. Previous studies on the LBS of shoes have mainly focused on the variations in the ankle and MTP joints, with relatively few studies on the hip joint (20). The present study has observed that higher LBS resulted in a reduction in hip extension angle, consistent with the results of Chen (36) et al. that exhibited a reduction in hip ROM under HLBS conditions. They attributed the reduction in hip extension angle to inadequate musculoskeletal development and immature motor control systems in adolescents. The difference between these studies lies in the age of the participants. They recruited adolescents, whereas the subjects recruited for the present study were adult males. Nevertheless, the participants did not show significant differences in hip mobility during the bracing period, but only in maximum hip extension angle. Adult runners, especially those who regularly participate in running training, are more experienced and usually have a more stable gait. They can also effectively cope with the effects of changes in sole stiffness, exhibiting lower joint ROM and greater joint moments than novice runners (37). Therefore, the reduced hip extension angle observed in this study was primarily due to the rigidity of the high LBS shoes, limiting hip extension.

The analysis showed that, compared with LLBS, HLBS shoes significantly reduced the negative work at the hip and MTP joints. These reductions in mechanical energy absorption may help improve energy efficiency during running. This finding is consistent with that of Oh (8) et al., who explored the effect of sole stiffness on the natural bending angle of the MTP joints. They suggested that a moderate increase in the LBS has the potential to easily reduce the energy consumption of running. Moreover, the positive work of the ankle joints increases. This directly supports our initial hypothesis that increased LBS would reduce negative work at the MTP joint while increasing positive work at the MTP and ankle joints. Sun et al. (24) similarly observed that increasing LBS was associated with reduced peak dorsiflexion angle and negative work at the MTP joint, which aligns with the current findings. However, Sun et al. also reported a reduction in the relative positive work of the knee joint under HLBS, the result that differs from our finding that both positive and negative work at knee joint showed no significant differences across the three LBS conditions. The reason for this biomechanical difference may be due to the different placement of the CFP. Flore et al. (28), in investigating the effect of carbon plate placement on lower extremity biomechanics, found that the knee positive work was reduced with the high placement of the CFP compared with low placement. Cigojia et al. (38) attributed the increase in positive work at MTP joint to the earlier timing of MTP plantarflexion, combined it with a larger peak MTP moment, resulting in higher MTP joint positive work and a higher metabolic cost during running. This conclusion, however, is contrary to numerous studies showcasing that increased LBS favors runners to run more economically (13, 37). Another possible explanation is that the CFP functions like a spring, storing and releasing elastic energy as the MTP joint undergoes dorsiflexion and plantarflexion during the stance phase of running (37). The increase in the positive work from the ankle joint, may be due to the increase in the LBS, limiting the dorsiflexion activity of the MTP joints. As a result, an anterior shift of the plantar touchdown point occurs during the stance phase, thereby altering the point of action of the GRF and lengthening the force arm of the ankle joint (39). Muscle-tendon units around the ankle joint are more capable of storing and returning elastic energy, contributing to lower metabolic costs during long-distance running (38). Uchida et al. (40) demonstrated through musculoskeletal simulations that compliant tendons, such as those of the soleus, allow muscle fibers to operate nearly isometrically during stance, thereby reducing metabolic power consumption. The increase in the ankle positive work implies that runners generate more effective forward propulsion through the ankle joint, reducing the reliance on thigh and gluteal muscle groups (e.g., quadriceps and gluteus maximus), as well as the burden on the hip and knee joints.

The enhancement of LBS of shoes is not simply linearly related to running performance. This study showed that although the negative work at the hip joint was significantly lower in the HLBS running shoe condition than in the LLBS shoe, the maximum joint moment was significantly higher than in the MLBS condition. This suggests that an increase in joint moment does not necessarily imply an increase in joint work. The larger hip joint moments observed in the HLBS condition may reflect a rise in resistance due to restricted joint movement, which in turn decreases angular velocity and shortens the duration of energy absorption. This increase in transient mechanical loading may increase the muscular demands on the peripheral hip muscle groups, particularly the gluteus maximus and hamstrings, during prolonged running or lead to the early onset of fatigue and even increase the risk of overuse injuries. It has been noted that greater moment loads on the proximal joints not only increase metabolic costs, but may also induce the development of compensatory movement patterns (41). Compared with HLBS, MLBS preserved greater MTP joint ROM and dorsiflexion range while also resulting in lower hip joint moments. This suggests that MLBS achieves comparable energy-saving effects while maintaining better joint kinematics. Furthermore, MLBS induced lower hip joint moments than HLBS, potentially reducing the mechanical demand on proximal musculature. Therefore, MLBS may offer a more favorable balance between biomechanical efficiency and movement freedom, making it a more suitable choice for long-distance runners. This study focused on elite male runners with relatively homogeneous performance levels. Therefore, the generalizability of the findings to recreational runners, female athletes, or younger populations remains uncertain and warrants further investigation.

## Conclusion

This study demonstrated that increasing the LBS of shoes significantly affects the kinematic and kinetic characteristics of lower limb joints in elite rearfoot-strike runners. Specifically, higher LBS limited MTP joint dorsiflexion and reduced negative work at the hip and MTP joints. Although both MLBS and HLBS shoes exhibited similar reductions in energy absorption compared to LLBS, the MLBS condition better preserved MTP joint mobility and imposed lower mechanical loads on the hip joint. These findings suggest that MLBS provides a more favorable balance between biomechanical efficiency and natural movement freedom. Therefore, MLBS may be a more suitable choice for elite marathon runners and provide valuable insights for future optimization of LBS parameters in running shoe design.

## Data Availability

The original contributions presented in the study are included in the article/Supplementary Material, further inquiries can be directed to the corresponding authors.
